# Co-Designing an Intervention to Increase HIV Testing Uptake with Women from Indonesia At-Risk of HIV: Protocol for a Participatory Action Research Study

**DOI:** 10.3390/mps2020041

**Published:** 2019-05-23

**Authors:** Corie Gray, Gemma Crawford, Roanna Lobo, Bruce Maycock

**Affiliations:** Collaboration for Evidence, Research and Impact in Public Health, School of Public Health, Curtin University, 6102 Bentley, Australia; G.Crawford@curtin.edu.au (G.C.); Roanna.lobo@curtin.edu.au (R.L.); B.Maycock@curtin.edu.au (B.M.)

**Keywords:** HIV, migrants, culturally and linguistically diverse, participatory action research, co-design

## Abstract

Early diagnosis is a critical component of the global response to the human immunodeficiency virus (HIV). In Australia, more than two-thirds of women from Southeast Asia are diagnosed late with HIV. There is limited evidence regarding the barriers to HIV testing and which interventions work to increase an uptake among migrants living in high-income countries. This participatory action research (PAR) project will work with women from Indonesia to co-design an intervention to increase HIV testing uptake in Western Australia. The project will involve trained community researchers, representatives from relevant organizations, and community women born in Indonesia. We will conduct three PAR cycles. Phase one will use focus groups to understand enablers for HIV testing among community members. In phase two, data will be presented back to members of the participating communities who will be invited to co-design an intervention to increase HIV testing. The final cycle will focus on implementing and evaluating the resulting intervention. This project will add to the small body of literature on pathways and enablers to HIV testing, and to new insights regarding interventions that work for women from migrant communities and why.

## 1. Introduction

In the last decade, notifications of human immunodeficiency virus (HIV) in Australia have increased among people born overseas, predominately from Northeast Asia (NEA) and Southeast Asia (SEA) [1,2]. This growth can, in part, be attributed to increasing migration and mobility rates [3]. 

Global migration has been rapidly increasing, from 173 million in 2000 to over 258 international migrants in 2017 [4]. In Australia, in 2017, 29% of the population were born overseas, having increased from a quarter (25%) in 2007 [5]. There are a number of reasons for overseas migration, such as family reunion, travel and recreation, employment, education, or to escape war or conflict [6,7]. It is complex and dynamic, and for many migrants, multi-directional, with migration increasingly recognized as a global public health priority [8]. 

Migration can influence health status, with migrants facing significant intersecting health and social inequities [6,9]. Circumstances of migration, alongside challenges of resettling, may have an adverse impact on both mental and physical health [10]. Stigma and discrimination, unfamiliarity with the new culture, language skills, and legal status may also impact health outcomes [10,11]. As such, migrants face different challenges, in comparison to the host population, with certain groups being more vulnerable to HIV acquisition [12,13]. 

This vulnerability to HIV is linked to broader structural influences, such as poverty, gender inequality, and access to health services [12,13]. For recently arrived migrants, risk behaviour and access to health services and social support may change in the new country, increasing the vulnerability to acquisition [12]. Migrants face complex issues in regards to prevention, including differences in HIV knowledge, cultural beliefs and norms, attitudes towards condom usage and use of condoms, HIV-related stigma, and attitudes towards HIV testing [12,14,15,16,17,18]. Migrants may also continue to travel back and forth to their country of origin, where HIV prevalence may be higher and support for HIV prevention and testing differs [18,19,20]. Culture and gender norms may also impact vulnerability [21], with migrant women citing difficulties in negotiating condom use and difficulties accessing sexual health services without a partner [22,23,24,25]. 

For women from SEA living with HIV in Australia, almost two thirds (63%) are diagnosed late (measured by a CD4 cell count of fewer than 350 cells/µl at diagnosis), meaning they have lived with the virus for four or more years before diagnosis [2]. In comparison, less than a third (29%) of Australian-born women have a late diagnosis [2]. Late HIV diagnosis increases the risk of onwards HIV transmission, the likelihood of subsequent morbidity and mortality, and increases in health care costs [26]. Knowledge of personal HIV status allows for access to treatment, and is a critical component of Australia’s response to HIV.

Culturally and linguistically diverse (CaLD) people from high HIV prevalence countries (a high HIV prevalence country refers to a prevalence above 1% in adults aged 15–49 years [27]), are a priority population in Australia’s Eighth National HIV Strategy 2018–2022 [27]. In Western Australia, people born in Indonesia had the highest number of notifications among people born in SEA between 2011–2017 (unpublished data supplied by Department of Health Western Australia Communicable Disease Control Directorate). Half were heterosexual females.

Despite being recognized as a priority population for Australia’s HIV response, investment in health and support services for migrant populations, nationally, has been described as a ‘one size fits all’ approach [3]. Little information is available on how to increase testing among migrants living in high-income countries, with limited evidence of barriers and enablers to sexual health and HIV testing [23,28], and which interventions work [29]. Migrants are not a homogenous group, and even amongst those from the same country of birth there are differences in migration experiences, culture, race, gender, class, and age [30]. The need for more nuanced interventions to increase knowledge, improve access to services, and to increase HIV testing has been stressed from those working in the sector [3,20,23,31,32].

The involvement of migrant communities in both research and health interventions may be challenging [33], particularly so, for topics such as sexual health and HIV, which have been acknowledged as sensitive, culturally taboo, and highly stigmatized for some ethnic groups [34]. Migrant communities may be marginalized for a number of reasons, including discrimination and racism [9,33]. In addition, some migrant communities may feel ‘blamed’ for HIV, due to media focus on their communities [34,35,36]. Migrant women experience a number of intersecting factors that influence their health, including gender inequalities, cultural norms, and lack of social support, which may increase difficulties in accessing appropriate resources and services [37]. With the difficulties for migrant communities, in particular women, and the experience in accessing culturally acceptable health interventions and services [33,38,39], it is critical that they are active participants in research and prevention efforts [40].

This project will work with Indonesian women in Western Australia to develop a multi-strategic intervention to increase HIV testing, and further explore the intersection between migration and HIV [9,15,18,41,42]. The project will employ participatory action research (PAR) methodology to ensure the target population is actively involved. PAR is a combination of both research and social action. It has been defined as “systematic inquiry, with the collaboration of those affected by the issue, for the purposes of education or effecting social change” (pg. 174) [43]. PAR seeks to understand real world problems and develop solutions in collaboration with participants and stakeholders, exploring relationships and local contexts that pre-exist, empowering individuals, and advocating for change [44,45,46]. PAR puts the community at the centre of the research, allowing them to determine priorities and shape outcomes, and has a focus on conducting reflexive research that enables action [40,44]. 

A co-design process will be used to develop the intervention and evaluation. Co-design is a collaborative process, in which participants take an active role in the design of the intervention alongside academics and other relevant stakeholders [47]. The aim of co-design is to create a more satisfactory intervention and increase the likelihood of success and support of the innovation through the active involvement of the community and organisations [48]. Co-design is utilized in participatory methodologies, such as PAR [49], as it emphasises community empowerment and ownership [47,50,51]. 

## 2. Aims and Objectives

This research will use participatory action research methodology to co-design an intervention to increase HIV testing uptake among women from Indonesia in Perth, Western Australia (WA). The objectives of this study are to:

- Identify and assess the pathways and enablers to HIV testing among women from Indonesia
- Record and synthesize the use of a co-design process in developing an intervention
- Determine the critical elements of a co-designed intervention to increase HIV testing uptake among women from Indonesia
- Implement and evaluate a co-designed intervention to increase HIV testing uptake among women from Indonesia
- Review the utility of a participatory action research approach in addressing HIV with women from Indonesia
- Synthesize findings of the intervention and provide recommendations on appropriate strategies to prevent and manage HIV among women from Indonesia.

## 3. Methods and Analysis

There is no fixed process for the design and implementation of PAR studies, nor is there a singular theoretical framework [52]. Instead, PAR is context-specific, with the process driven by participants and requiring innovative methodological approaches [53,54]. The underlying ontology of PAR believes “that human beings are dynamic agents capable of reflexivity and self-change” (pg. 72), with an epistemology approach that “accommodates the reflexive capacities of human beings within the research methodology itself” (pg. 72) [54]. This approach allows for participants to explore and reflect on their own beliefs, generate and analyse data, and determine necessary action [32,54]. 

**Figure 1:**
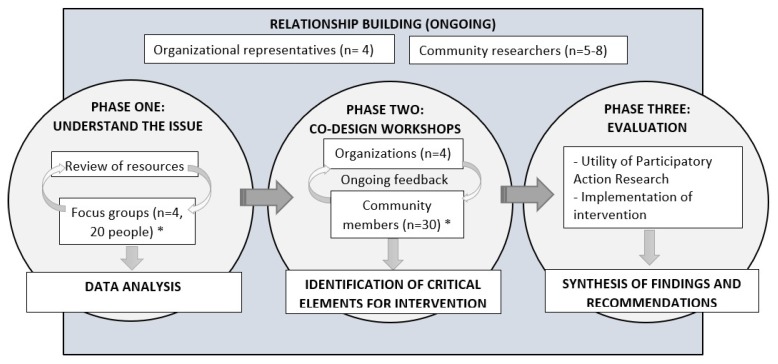
Project flowchart. Note: The above flowchart shows the minimum numbers required (indicated by *). Cycles of participatory action research will continue until it is felt that there is sufficient understanding to progress to next cycle.

Three cycles of PAR will be conducted (Figure 1). PAR is a framework with a “cyclical process of fact finding, action, reflection, leading to further inquiry and action for change” (pg. 191) [55]. The first phase will use focus groups to understand enablers for HIV testing among community members. Phase two will involve synthesized data for member checking and co-design of an intervention to increase HIV testing. Finally, the intervention will be implemented and evaluated. 

### Partnerships

There will be three groups of participants in this study: (1) Peer community researchers, (2) representatives from relevant organizations, and (3) women from Indonesia (referred to as community members). Involving both community researchers and organizational representatives will improve understanding of the issue, increase ownership at both a community and organizational level, as well as integrate knowledge into organizations to improve practice and increase the sustainability of the intervention after the project has finished [46,56]. 

Organizations represented will include the state Department of Health and the state peak organization for HIV. It may include additional organizations relevant to Indonesian women identified in phase one. Organizations will assist to establish relationships with community members, inform the development of the intervention and collaborate in the implementation and evaluation of the intervention. Between five and eight Indonesian women will be trained as community researchers for the life of the project. Recruitment will occur through existing networks of organizations, with community researchers purposively selected based on their English proficiency, and connectedness to community. Representation will be sought from individuals from different age groups, relationship status, educational backgrounds and length of time in Australia. This will ensure that community researchers involved will be able to connect with people from a range of backgrounds. The Eighth National HIV Strategy [27] outlines as its first guiding principle the centrality of people with HIV and the meaningful involvement of priority populations [57]. Along with participation of women from Indonesia as a priority population, representation will be sought from a person living with HIV who may provide valuable insights about the factors which influence testing and late diagnosis.

Common to PAR, community researchers will be involved throughout the project [37]. Community researchers bring unique experiences and perspectives to the research. Community researchers will help to articulate the experience of research participants and share insights from the community perspective, overcome language barriers, provide a link between community members and researchers to help build trust, and influence research to ensure appropriate methods and representation of community [37,58]. It also aligns with PAR principles of doing “research with” community [37]. 

Training will be provided to community researchers by the research team on HIV and sexual health, project governance, and relevant research skills. This will help build the research capacity of the group and facilitate community ownership of the project [59]. The group will facilitate access to the broader community and assist in the co-design, implementation, and evaluation of the intervention. They will also take an active role in the development of data collection tools, data collection (through co-facilitation of focus groups and workshops), and data analysis. Community researchers will be volunteers, and will be acknowledged with a small honorarium, opportunities to access training, the provision of food at research meetings, out of pocket expenses reimbursed (e.g. parking charges), and by specifically acknowledging community researchers (with consent) in the dissemination of findings.

## 4. Phase One

This phase will include establishing community researchers, conducting a review of HIV resources, and focus groups with community members. An extended time-period (approximately 12 months) has been planned to allow for the further development of relationships between the lead researcher and community, critical to the process of PAR. 

### 4.1. Review of Resources

During phase one, HIV resources from both Australia and Indonesia (when available in English) will be reviewed. An online search for available resources will be initially conducted via www.allgood.org.au—a national website housing information on sexually transmitted infections (STIs) and bloodborne viruses (BBVs) for both culturally and linguistically diverse and Aboriginal and Torres Strait Islander communities. Further online searches will be conducted to identify relevant resources. 

The Health Literacy INDEX tool will be used to evaluate the health literacy demands of identified resources [60]. INDEX is comprised of 63 indicators organised into 10 criteria, including audience appropriateness, evaluation methods, and strength of evidence [60]. A content analysis will be undertaken to determine the type of information included, such as information regarding modes of HIV transmission; incorporation of strategies to reduce risk of acquisition; and emphasis on testing and treatment [61,62]. This process will explore how messages about HIV and AIDS are developed and conveyed to migrants living in Australia, and further inform the design of the intervention in phase two.

### 4.2. Focus Groups

Purposive sampling is the deliberate selection of individuals who can provide in-depth relevant information as well as broader views [63]. Organizational representatives and community researchers will purposively recruit eligible community members to participate in focus group discussions. Eligible participants will be aged 18 years or older, female, born in Indonesia, and have been in a sexual relationship. Twenty participants will be involved across four focus groups, with 10 people who have previously voluntarily tested for HIV in Australia and 10 who have not. Further focus groups will be conducted if needed, and participants may be invited to take part in follow-up interviews, to further explore individual experiences of migration and pathways to HIV testing [15]. 

#### 4.2.1. Data Collection

A short survey will collect demographic data from focus group participants. Informed by literature, the focus group guide will be developed in collaboration with community researchers and organizational representatives. Relevant theoretical sources (selected based on discussions with community researchers) will inform question development and data analysis [18,21,41,64]. The focus groups will be co-facilitated with community researchers and explore migration experiences [15], health service access, enablers to HIV testing, and feedback on existing resources for HIV (as gathered in the review). These discussions will be audio recorded with consent from participants and each will be approximately one and a half hours in duration. 

Focus group discussions were chosen for their strengths in directly reflecting the voices of marginalized groups and the ability to encourage participants to share, build on, and disagree on sensitive topics [65]. Focus groups also help to validate points raised as ‘shared’ experiences, through the synergistic effects of discussion and contribution of ideas [66,67]. Focus groups may also enable people with low literacy to participate; can encourage participants who may be reluctant to be interviewed on their own; and give insight into the groups’ lived experiences [66]. Previous research has used focus groups with other culturally and linguistically diverse communities in Australia, including in sexual health research [35,68]. 

Community members will describe and draw maps of their interactions with health services and other sources of help-seeking (i.e., multicultural organizations with information on sexual health, religious leaders, websites, etc.), and perceived pathways and enablers to testing for HIV [69,70]. Community mapping will collect both the output (maps), plus the verbal descriptions of each element of the map for analysis. Feedback on different relevant resources (from both Australia and Indonesia) will be collected during the focus groups. Type of feedback will include: Relevancy of the content to community, usefulness of the material, and the types of images and language used, as well as what is missing. 

#### 4.2.2. Data Analysis

Audio recordings of the focus groups will be transcribed verbatim by the lead author enabling data immersion [71]. Qualitative data will be analysed thematically [71] (consistent with other PAR studies) [72] and coded using NVivo 11 software [73]. Thematic analysis is a method for identifying patterns of meaning (or themes) within a dataset [71]. The six phases of thematic analysis identified by Braun and Clarke, 2006 will be used [71]. Initial coding will be directed by relevant theories and research questions identified by the research team and community researchers. Codes will be collated into potential themes, using a process known as ‘describe-compare-relate’ [74]. Potential themes will be discussed and refined with co-facilitators and community researchers and with the organizational representatives [75]. 

Community maps will be retained as physical copies, as well as captured electronically. This will involve describing the images used and linking it to the participant’s descriptor of the image. Data will be analysed thematically [71], using the same process as focus groups. 

## 5. Phase Two

Due to the nature of PAR, the complete scope of the second and third cycle will be unknown prior to the completion of phase one. The second stage will be to plan an intervention to increase uptake of HIV testing. It will be community-driven through co-design workshops [19,56,76] and reflect the needs, preferences, and knowledge gaps of Indonesian women (broader community), with input from relevant organisational representatives, community researchers, and the research team. A summary of data collected in phase one will be presented back to community members during co-design workshops and inform subsequent discussions. Co-design is an iterative process [47], and workshops with community and feedback with organisational representatives and community researchers will be ongoing to determine critical elements of an intervention. This intervention will have multiple approaches or activities, known as “strategies”. 

Selected strategies that can be implemented within the time of the project will be identified in initial discussions (2 to 3 strategies are likely to be trialled). These strategies may aim to develop relevant skills within the community to access services, reduce HIV-related stigma, and encourage uptake of HIV testing. Where possible, strategies will build on the existing strengths and opportunities within the community, such as religious leaders or existing community events, and within organizations. This will also involve determining the expected process, impact, and outcome evaluation indicators of a successful intervention with community and organization representatives.

Previous examples of strategies arising through PAR include: Educational workshops delivered by peers [77], advocating for change through report-writing, letters, and meetings [78,79,80], peer-based education, and the development of health promotion or educational materials [81]. Other potential interventions may include: Outreach HIV testing and increased offers of testing by GPs, and media campaigns [29]. Appropriate health promotion theories will inform the development and implementation of particular strategies. 

### 5.1. Sampling Strategy and Participants

Workshops will be conducted with community members to ensure a variety of ideas are heard and to bolster ownership from community. Relevant organisational representatives and the research team will be invited to provide feedback on the identified strategies. 

### 5.2. Data Collection and Analysis

Community researchers will co-facilitate all events. Workshops will be ongoing until final strategies have been decided on. Notes will be taken during workshops, and in subsequent meetings with community researchers, organizational representatives, and the research team to provide an audit trail of decision-making. Data will be analysed thematically [71], though the focus will be on action and the development of strategies. 

## 6. Phase Three

In partnership with community and organisational representatives, selected intervention strategies (identified in phase two) will be implemented and evaluated. It is likely that strategies will only reach proof of concept, or be tested for feasibility [82,83] among a small group or setting. Evaluation of the intervention will assess implementation outcomes discussed by Proctor et al, including early indicators of appropriateness, acceptability, adoption, feasibility, and implementation cost [84,85]. The involvement of organisations in the design of the intervention will support ongoing changes in practice, and increase the sustainability of the intervention after the project has finished.

Over the course of the PAR project, a number of methods will be used to record and review the utility of both the PAR approach and co-design element [86,87], the experiences of community researchers [88,89], and the implementation of the intervention [84,85]. This will include feedback from community members, community researchers, and organisation representatives through interviews, focus groups or other culturally appropriate evaluation strategies (e.g. photovoice [90]) and researcher reflection [90,91,92,93]. Quantitative data, including surveys and observation, may also be used [81,94]. 

### Sampling Strategy and Participants

The intervention will target community members, and a subset (not involved in the co-design) may be involved in the feasibility testing of the intervention. Evaluation of the process of the research will include community researchers, community members involved in the co-design process and organisational representatives. 

## 7. Ethical Considerations

This project has ethical approval from the Curtin University Human Research Ethics Committee (HRE2018-0790). Informed consent will be obtained from participants prior to taking part in focus groups in phase one, and potentially for evaluation in phase three (depending on the type of evaluation used, i.e., interviews, surveys, focus groups). Informed consent will also be obtained from community researchers. 

To address ethical concerns in working with culturally and linguistically diverse populations, the “4 Ps” framework to ensure culturally safe research will be applied: Partnership, participation, protection and power [40]. This framework further supports the use of PAR, which addresses all components of the 4 P’s. It provides further guidance on the importance of critical reflection of beliefs, attitudes, power relationships, and approaches to research [40]. 

Community researchers will facilitate the inclusion of non-English speaking participants, including translating participant information sheets and consent forms (where relevant), to ensure participants understand the nature of the research. We anticipate that most participants will speak English. 

## 8. Conclusions

The results of the project will further add to the literature on what interventions work with migrant communities and why. It is likely to have relevance to other jurisdictions, and other community groups, as a process of working with community. This work is in line with the Australian Government’s goal of virtual elimination of new HIV transmissions by 2022 [27], with a focus on a population that, whilst mentioned as a priority population in most of the national strategies over the past three decades, to-date has not been at the forefront of Australia’s response to HIV.
